# Lectures to Nurses

**Published:** 1907-05-11

**Authors:** 


### LECTURES TO NURSES.

We have received, from a correspondent signing himself "C. E. S.," an amusing communication on the subject of lectures by house surgeons to nurses. The title of his article is "Nurses' Howlers," and we regret that space forbids us to publish it in its entirety.

The house surgeon "may spend many hours preparing his lectures, and may deliver them in the simplest and plainest of language; but the result—as shown by the inevitable written examination—is always disappointing. Especially is this true when he is trying to describe the position and relations of the various internal organs. His only help, in many cases, lies in the black board, and his own skill with coloured chalks thereon. . . . He may think, after taking unusual care over a lecture, that his audience must have a perfect grasp of the matter; but let him ask a question or two about it, and his self-esteem as a good lecturer receives a rude shock."

Our correspondent goes on to quote several answers to questions which caused him, to use his own words, "pained amusement." We feel constrained to print three examples, culled from the domains of anatomy and medicine.

"The ileo-cæcal valve is situated at the heart, and keeps that organ supplied with blood necessary to enable it to carry on its functions."

"When the temperature comes down by crisis the patient dies suddenly, when by lysis it shows internal hæmorrhage."

From a ward night report: "Mrs. S—— had a comfortable night; did not require a ward-pill; died at 4 a.m."

We appreciate the difficulties that lie in the way of giving elementary instruction in anatomy, physiology, and medicine to those who have had no previous training in chemistry, physics, and biology; and we sympathise with the lecturer in his disappointment at the apparent results of his teaching. But, however little his pupils may seem to profit by these lectures, we are convinced that the revision and simplification incidental to their preparation and delivery is of much value to the lecturer himself. The house surgeon is in this way compelled to refresh his memory with many facts and principles of the highest importance, which, but for the stimulus of these lectures, he is only too prone to forget.

Moreover, there is little doubt that the position of teacher, in addition to that of superior officer, strengthens the authority of the house surgeon, and tends to increase the cordiality of his relations with the nursing staff.

